# Assessment of the Materials Employed in Green Artificial Reefs for the Galician Estuaries in Terms of Circular Economy

**DOI:** 10.3390/ijerph17238850

**Published:** 2020-11-28

**Authors:** Luis Carral, Carolina Camba Fabal, Mª Isabel Lamas Galdo, Mª Jesús Rodríguez-Guerreiro, Juan José Cartelle Barros

**Affiliations:** Escola Politécnica Superior, Universidade da Coruña, 15403 Ferrol, Spain; l.carral@udc.es (L.C.); carolina.camba@udc.es (C.C.F.); isabel.lamas.galdo@udc.es (M.I.L.G.); maria.guerreiro@udc.es (M.J.R.-G.)

**Keywords:** artificial reef, circular economy, renewable resources, organic residues, mussel industry, sustainability

## Abstract

To exploit marine resources in a sustainable way, efficient management systems must be used such as green artificial reefs (GARs). These reefs are mostly made up of renewable and organic materials. When adopting the circular economy (CE) model, industrial processes must be reconsidered. By adapting how conventional artificial reefs (CARs) are engineered and produced to embrace the principles of the CE, certain materials can be used. Renewable resources are designed to be reintroduced into the biosphere without producing harmful organic residues or nutrients. Within a framework that covers economic, environmental and social considerations, this study offers four new proposals related to substituting the materials destined for the components in an artificial reef. For the first time, two different methodologies were applied to determine the best alternative in terms of its contribution to both sustainability and CE. From the results obtained, the best solutions are in line with substituting a certain amount of the cement and sand with mussel shells. The importance of the results lies in the fact that the canning industry in Galicia (northwest Spain) generates shell residues which promote grave environmental consequences.

## 1. Introduction

### 1.1. The Role of Green Artificial Reefs in the Circular Economy

Across the world, the natural habitats of coastal ecosystems are seriously threatened due to global climatic change and marine pollution. Overly intense activity in fishing grounds is another factor to consider [1,2,3,4,5,6,7]. Initiatives like fish farming and creating artificial reefs might mitigate the situation in which fishing resources are dwindling [8,9,10,11,12].

Artificial reefs (ARs) create suitable habitats for marine species if the following design factors are taken into account: depth [13], water circulation [14], and materials employed [15,16,17,18]. These reefs provide additional surfaces, hollow spaces and clefts into which organisms can settle [19,20]. They also serve as a rich food source for herbivores and as a refuge for many species, including large non-reef inhabiting predators [21,22,23,24]. Moreover, the complexity of this habitat attracts a wide variety of fishes [25]. Any kind of artificial structure that shelters fish and other sea organisms provides a habitat that was inexistent before its construction [26].

Opportunity artificial reefs (OARs) are mainly: (i) out-of-service vessels that have been sunk; and (ii) re-used tires. In fact, several authors have studied the way in which objects are cast into the sea to improve fish habitats [27,28,29]. Old fishing vessels deliberately sunk can also serve to provide further points of interest for recreational scuba diving [30,31]. In this line, Devault et al. [32] consider using deliberately sunk vessels to create ARs as an alternative to their land-based recycling.

In general, the concept of sustainable development involves using natural resources in a controlled way as well as reducing energy consumption, and with it, the emissions of greenhouse gases. Despite ecological measures, world ecosystems are increasingly under pressure [33] reaching their global limits [34,35]. This trend shows that both sustained economic growth based on a linear production model and unsustainable consumption patterns are unfeasible on a planet with finite resources and a limited capacity to absorb waste [36].

**Figure 1:**
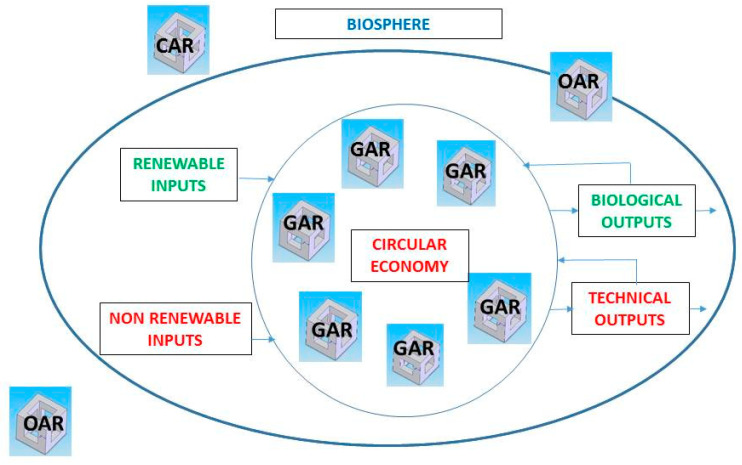
The adequate size of an economic system and types of viable artificial reefs. GAR: green artificial reef; CAR: conventional artificial reef; OAR: opportunity artificial reef.

For these reasons, the circular economy (CE) has been put forward in the last decade as a general concept [37]. The CE is the alternative to the traditional linear economy (make, use, dispose), in which the resources are re-used or recycled at the end of each service life. Since the scientific literature about CE remains scarce [38], there is ample debate about how to implement its principles [39,40,41]. The CE envisions an economic system in which resources are more efficiently exploited; the way in which energy and material flow is intentionally closed and reduced [42,43]. In this context, Suarez-Eiroa et al. [38] consider that, within the sustainable development framework, the objective of the CE should be towards reducing the economic system to proportions acceptable to the biosphere [44]. Green artificial reefs (GARs) are in accordance with the policy of this reduced framework. Consequently, Figure 1 illustrates the adequate size of an economic system according to CE. The oval represents the planetary boundaries, which are being transgressed by the effects of the anthropogenic damage on the global environment. The main principle of CE consists of closing and reducing the flows of energy and materials, represented through the circle. Contrary to conventional artificial reefs (CARs), green artificial reefs (GARs) are included in this limited field. In fact, the inclusion of artificial reefs inside the biosphere depends on its typology (CAR, OAR or GAR). The materials employed in GARs must derive from renewable inputs, represented in green colour in Figure 1. These materials must be reintroduced into the biosphere without incidents, as biological outputs. This is equivalent to reducing or eliminating technical outputs (wastes), represented in red colour in Figure 1. Contrary to technical outputs, biological ones are biodegradable and can flow through cycles.

Among CARs, concrete is the construction material that is most commonly used for large and hollow modular structures [16,45]. Concrete is mainly made from a mixture of cement, sand, gravel and water. In terms of sustainability, it would be desirable to replace cement and other dry materials by renewable resources. This may also lead to a reduction in costs. In addition, the cement industry constitutes one of the greatest industrial sources of carbon emissions [46].

In pursuit of sustainability and with the objective of benefiting current and future generations, a balance is needed between economic results, social responsibility and environmental commitment [47]. Building ARs with waste materials combined with reinforced concrete [15,48], may be of interest, but only if the materials added to them are environmentally friendly [29]. GARs rely on renewable material flows designed to be re-introduced into the biosphere without repercussions. Consequently, it is now necessary to check if they are real feasible options by assessing their real contribution to both CE and sustainable development, by using scientific methods. This was the main objective of this study. Table 1 shows the potential effectiveness of different types of ARs at the time of meeting economic, social and environmental objectives. These potential results must be verified from a scientific point of view.

In any case, the transition from CARs to GARs would be in keeping with the idea of balancing the interface between the economy and the environment, that is, the precept of the circular economy (CE) [49]. Now that the era of cheap materials promotes the linear economy (make, use and dispose), a more coherent model must be implemented [38]. The present work aimed to demonstrate if the transition from CARs to GARs is beneficial for the Galician estuaries. Since the canning industry in Galicia generates thousands of tons of shells, this residue may be a good alternative for substituting some of the conventional materials used in concrete. Furthermore, another potential course of action is to use eucalyptus fibres as an alternative material for the conventional reinforcement of concrete. As will be explained later, these two possibilities were considered in this study. Section 1.2 provides a state of the art overview about the canning industry in Galicia and the possibility of employing mussel shells in the composition of concrete. Section 1.3 provides a state of the art overview about the eucalyptus industry in Galicia and the possibility of employing eucalyptus fibres as frames for reinforced concrete.

### 1.2. The Canning Industry in Galicia

Seashells are among those waste materials that accumulate rapidly, especially in coastal zones and areas of high seafood consumption. In this context, the Galician canning industry is the second biggest one in the world [50]. In fact, aquaculture and the canning industry are two very important sectors of the Galician economy, accounting for the creation of thousands of jobs. On the other hand, the industrial process of cooking mussels for canning, freezing or food preparation processes generates a significant annual volume of shell residues. This constitutes a serious environmental problem since shells residues usually end up in controlled solid urban waste containers, containers authorised by Public Administration, in shellfish processing companies or in uncontrolled containers, the latter due to the difficulty of the daily disposal of shell waste in a high consumption season [51]. To this should be added the illegal dumping of mussel shells, which has important environmental consequences. First, it produces strong smells caused by the decomposition of organic material attached to the shells through the synthesis of volatile and toxic amines which give rise to air pollution [52]. Moreover, the organic matter in the decomposition process is colonized by microorganisms which could, through insects, be transmitted to animals and humans [53].

Secondly, the high concentration of calcium carbonates in shells causes a high quantity of Ca^2+^ ions to be produced, which combine with the leachate waters in ground surfaces, causing the eutrophication of lakes and estuaries, in turn giving rise to serious mortality problems. This also negatively affects living conditions in the sea by means of a sediment reduction [51]. The problem is of such magnitude that in the law relating to solid urban wastes in Galicia, 10/1997, 22 August, shells processed for the canning industry are considered harmless industrial waste. This is the reason why Galician companies are being set up to process various bivalve shells (mussels, oysters, clams…) with the objective of generating valid products in distinctive areas: soil fertilizers [54], water sewage treatment, gas purification and mineral elimination [55,56], as we as the collection and manufacture of various compounds [57].

**Figure 2:**
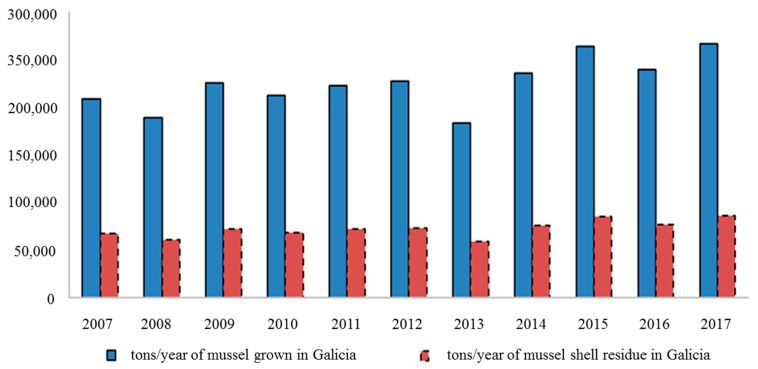
Evolution of mussel farming and shell waste in Galicia from 2007 to 2017. Tons of waste is calculated on the basis of a 32% bivalve shell weight.

Another point of interest is the possibility of using shells as building materials, partially replacing cement and sand. In this regard, bivalve shells can be used as an aggregate constituent in artificial reefs [16]. Since mussel farming is the most important and biggest waste generator of all of Galicia’s bivalve farming industry (Figure 2), there is a particular interest in the use of this type of shells. In fact, according to ANFACO (Asociación Nacional de Fabricantes de Conservas de Pescado, or National Association of Tinned Fish Manufacturers, in translation), the Galician canning industry produced 85,412 tons of mussel shell waste in 2017 [58].

The adaptation of the Management Plan for Solid Urban Waste in Galicia [59] established three basic principles of waste management in accordance with European Union directive management criteria:

- Minimise waste production;
- Maximise waste exploitation;
- Safe disposal of non-exploitable waste.

Consequently, the possibility of incorporating mussel shells to build green artificial reefs was analysed in this study. This work continues the research carried out during the PROARR project, financed by Xunta de Galicia (Spain) to develop efficient ARs in the Galician estuaries.

### 1.3. The Eucaliptus Industry in Galicia

Cement, the most used material in civil construction, behaves erratically under varying stresses. It is characterized by high compressive strength but at the same time, it presents a very limited resistance to tensile forces. To overcome the latter disadvantage, cement is combined with an interior steel reinforcement, giving rise to a matrix which performs well under both situations, although it tends to crack in the future. Despite its effectiveness, the steel reinforcement is expensive and of limited duration [16]. It is also associated with considerable energy consumption.

Durability is a key factor in the application of reinforced concrete to maritime infrastructures, as its high permeability allows the penetration of aggressive elements such as water, which leads to carbonation and attack from chloride ions, causing corrosion problems [60,61]. The phenomenon of corrosion in steel bars is the principle cause of deterioration. In this regard, Gjorv [62] cites a study of Norway’s OPC (ordinary Portland cement) which “indicates that 25% of those built after 1970 show corrosion problems”, while Ferreira [63] mentions that “40% of the 600,000 bridges in the U.S.A are affected by corrosion problems and they estimate a 50,000 million dollar cost in repair operations”. Mora [64] indicates that “if we could increase its useful life from 50 to 500 years, its environmental impact would be reduced tenfold”.

Apart from the local reinforcement of concrete using steel, the possibility exists of using fibres intermittently and randomly distributed throughout the entire concrete matrix. The use of artificial and natural fibres has received widespread attention in recent decades on account of their effectiveness [65,66]. With regard to concretes and mortars, preference has been given to the use of artificial fibres (glass and metals) [67], in search of improving resistance to traction, flexion, fracture, impact and thermal shock [68,69,70], as well as resistance to wear and tear (durability).

In the use of fibres, the choice of artificial or natural is not the same as the production of artificial fibres such as poly vinyl alcohol (PVA) and polypropylene, which are not in the desired line of sustainability [71]. This represents an opportunity for some types of natural fibres, with similar mechanical properties, higher profitability [72] and by contrast, greater sustainability. By way of example, Pacheco-Torgala and Jalalib [72] show that “a way towards sustainable civil construction will be the use of concretes reinforced with vegetable fibres as these come from renewable sources and are available at a relatively low cost in comparison with artificial ones”. In a similar line, Onuaguluchi and Banthia [73] highlight that “the benefits of large-scale use of vegetable fibres as raw materials in concretes are important in terms of energy, resource and environmental conservation”. Mukhopadhyay and Khatana [68] add that “natural fibres are gaining importance over synthetic, a consequence of their indisputable advantages stemming from their low cost based on abundance, biodegradability, flexibility, low density, relatively high tensile and flexion modules, minimum health risks, etc.”

Among the available natural fibres used in concrete, Brandt [74] highlights those of vegetable origin for their availability, claiming that “incorporating vegetable fibres into cement presents a clear opportunity to work with a resource which is available almost worldwide”. Vegetable fibres correspond to natural compounds with a cellular structure with varying proportions of cellulose, hemicellulose and lignia [75], and include cotton, hemp, jute, linen, ramie, sisal, bagasse and special fibres processed from timber (eucalyptus, etc.).

Many studies have detailed the favourable properties of using vegetable fibres extracted from the waste of cheap, rapid-growth agricultural crops; coconut shell [76,77], sisal [78], sugar cane [79], bagasse [80], bamboo [81], awkara, banana and musamba [82].

All of the natural fibres show a high tensile strength and a low elasticity modulus. Their tensile behaviour can approach that of synthetic fibres, as shown in Table 2. As vegetable fibres show differing compositions, it is to be expected that their performance in a concrete matrix might differ according to those selected [65,83].

According to Mukhopadhyay and Khatana [68] and Bentur and Mindess [66], the use of continuous fibres as reinforcement increases the tensile strength, while a random distribution improves the tendency to crack. Khare [86] tried out various concrete beams with continuous fibres as reinforcement and concluded that bamboo can partially substitute the steel reinforcement. There are other studies in which the potential use of bamboo is analysed [72,87].

Although the application of vegetable fibres as a component of concrete is incipient, it can be seen as a great opportunity for GARs. In this line, the eucalyptus is currently one of the most relevant forest wood species, and one of the most widely projected in the Iberian Peninsula. Eucalyptus plantations represent only 20% of the forested surface of Galicia (Spain), a space which, nevertheless, produces over 60% of the wood produced in Spain [88].

This production makes the eucalyptus an important source of income in the Galician rural community. By way of example, in 2018, more than 35,000 Galician forest owners, individuals and mountain resident groups had a EUR 205,000,000 turnover, which corresponds to an annual tree-cutting figure of 5.9 million m^3^ [88].

### 1.4. Objectives of the Present Work

From the previous sections, it is clear that artificial reefs are necessary to mitigate the pressure that coastal ecosystems are currently suffering. Nevertheless, since ARs are aimed at generating a positive impact on the marine habitats, they must be designed and constructed by generating the least possible impact on the environment. In other words, if artificial reefs are not in line with the principles of the circular economy and sustainability, they can cause negative impacts as significant as those they seek to mitigate. Consequently, it is important to promote the use of alternative materials as potential substitutes for conventional ones. Therefore, one of the objectives of the present work was to propose potential changes in the composition of conventional artificial reefs, generating green alternatives. Taking into account the problems derived from the mussel shell residues generated by the Galician canning industry, mussel shells are proposed as substitute materials for cement and sand. Based on the principles of environmental sustainability, eucalyptus fibres are proposed as substitute materials for the steel frames of reinforced concrete due to its their availability in Galicia. Although this study is focused on Galicia, it can also be valid for other regions of the world with similar problems.

Four green artificial alternatives with different percentages of mussel shells and eucalyptus fibres are considered in this work. A conventional alternative was also analysed, since it constitutes a good reference for making comparisons. This is the first time that two different methodologies are applied to quantitatively assess how each one of the five alternatives contributes to both circular economy and the integral sustainability. The first technique was an analysis of materials flow. Under this method, the materials were characterised in terms of renewable and non-renewable inputs employed to build the artificial reefs as well as the technical and biological outputs at the end of their service life. The second method implies the creation of a multi-criteria decision model to assess the sustainability of each one of the artificial reefs, by taking into account economic, social and environmental factors. A model made of eleven indicators was constructed by the authors for such a purpose. To the best of the authors’ knowledge, there are no similar studies in the existing literature.

## 2. Methodology

Sustainable development as a general concept can be considered as vague and ambiguous. Thus, it becomes crucial to adopt scientific methods, previously used with different purposes, to measure the suitability of green artificial reefs, i.e., the “capacity” to adopt the principles of CE.

### 2.1. Substitution Proposals

**Figure 3:**
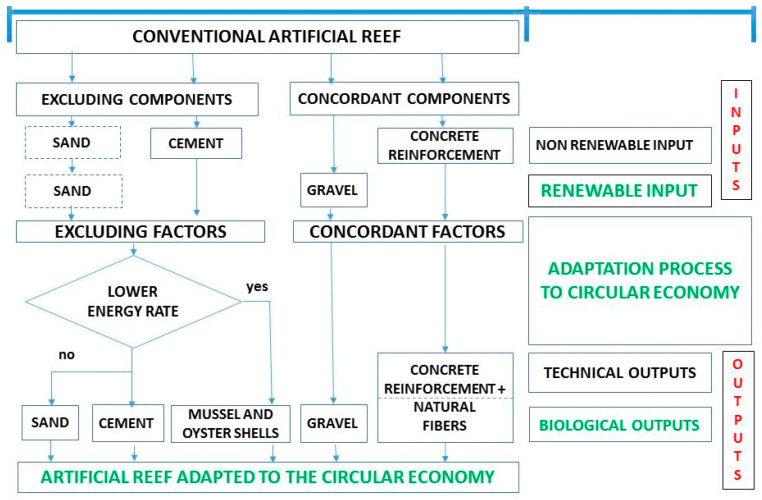
Decision tree that allows the transformation of a conventional artificial reef into an artificial reef adapted to the circular economy.

The idea of transforming a CAR into a GAR relies on the decision tree shown in Figure 3. With any industrial process, optimising sustainability in terms of component materials means limiting the consumption of resources and the emission of chemical residues. The first task involves maximising the exploitation of renewable materials within the limits of an ecosystem to replenish itself. The second task entails minimising any emission harmful to the environment. Figure 3 shows that there are two excluding components: sand and cement. These components can be substituted by seashells. Moreover, the reinforcement of concrete can be substituted by natural fibres as previously explained. Cement and usual reinforcements constitute non-renewable inputs, while sand and gravel are renewable inputs. Regarding the outputs, usual reinforcements can be classified as technical outputs while sand, cement, shells, gravel and natural fibres are biological outputs. A GAR adapted to the circular economy should include shells and natural fibres in its composition, trying to reduce both the extraction rate of non-renewable resources and the generation rate of technical waste through the use of substitute materials.

The CE places particular emphasis on activities that reduce, re-use and recycle materials [37]. Table 3 shows the CAR components and how to embrace this principle. Both in terms of product and residues, the level of their environmental impact can be considered low depending on whether renewable or non-renewable raw materials are used and if the residues are chemical or organic [89]. The last column includes the possibilities of applying CE principles. As can be seen, a substitution of cement, sand and metal reinforcement is proposed due to the environmental impact during production in the case of cement and metal reinforcement and during exploitation in the case of aggregates.

There are three common theoretical strategies within the paradigm of the CE:

- Minimising the input of raw materials and output of residues;
- Maintaining a resource’s value for as long as possible within the system;
- Reintegrating products into the system when they reach the end of their service life [90,91,92].

The reduction in sand and cement can be especially important due to the cement industry´s environmental impact in terms of carbon dioxide emissions (CO_2_), one of this industry´s by-products [46,93,94,95,96,97]. Cement manufacture consumes a lot of energy, requiring heating up to 1450 °C, resulting in a significant increase in its production costs [98]. The necessary resources for its production are becoming scarce worldwide; some countries already have problems acquiring them [99]. In any event, the global production of concrete increased notably in the last century and its future demand is expected to continue increasing [100].

Thus, the reduction in cement use in concrete production processes directly affects its reduction in emissions of CO_2_. Tayeh et al. [99] demonstrated that seashells constitute an adequate alternative for ordinary Portland cement since CO_2_ emissions from the cement hydration process are noticeably reduced.

Sand extraction is a mechanical action that harms our ecosystem [101]. Many studies have been carried out on the effects of seashells as addition replacements (fine and coarse) [102,103,104,105,106,107,108,109,110,111]. Carral et al. [16] propose incorporating crushed shells as an adequate substitute for 20% of the sand used in reinforced concrete. The possibility of substituting part of these metal frames which make up natural fibre-reinforced concrete (NFRC) is a highly interesting option which demands in-depth study [65,66,67,68,69,70,71,72,112].

Table 4 describes the following five proposals analysed in the present work:

- CAR: conventional artificial reef made by reinforced concrete (basically cement, sand, gravel and steel frame);
- GAR1: 10% of the cement is substituted by shells and 25% of the steel frame is substituted by eucalyptus fibres;
- GAR2: 20% of the sand is substituted by shells and 25% of the steel frame is substituted by eucalyptus fibres;
- GAR3: 5% of the cement is substituted by shells, 10% of the sand is substituted by shells and 25% of the steel frame is substituted by eucalyptus fibres;
- GAR4: 5% of the cement is substituted by shells, 10% of the sand is substituted by shells and 100% of the steel frame is substituted by eucalyptus fibres.

Table 4 also includes the dosage, i.e., the components of concrete (cement, sand, gravel and frames) presented in 1 m^3^ of artificial reef. The substitution percentages shown in Table 4 were taken from the existing literature. Tayeh et al. [99] reported that up to 10% of the cement can be substituted by oyster and mussel shells crushed and calcined at 600 °C. Regarding the sand, Carral et al. [16] concluded that 20% of the sand can be substituted by oyster and mussel shells crushed and baked at 300 °C. Regarding the frames, Mukhopadhyay and Khatana [68] reported that up to 25% of the steel frames can be substituted by vegetable fibres randomly distributed. Nevertheless, if some of these vegetable fibres are distributed in bars, the substitution can reach 100%. In this case, 25% can be randomly distributed but the remaining 75% of the vegetable fibres must be distributed in beams.

**Figure 4:**
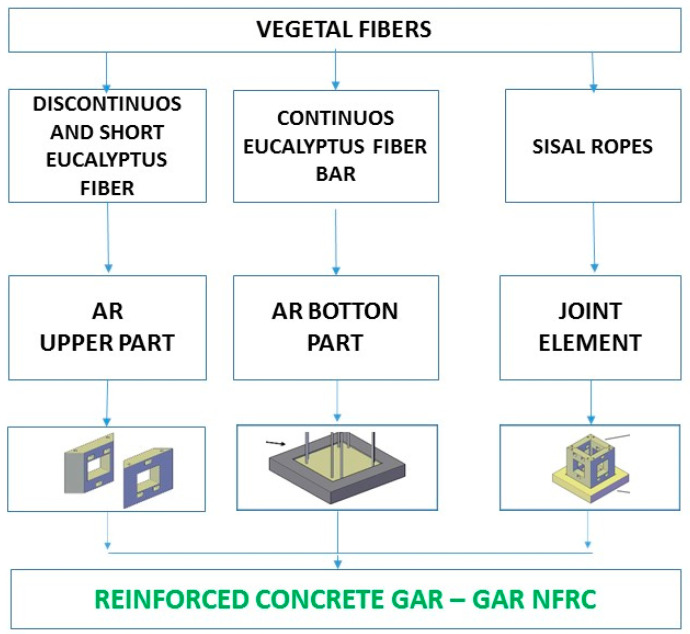
Detail of the concrete reinforcement at the bottom and upper parts of AR, using 100% vegetable fibres. GAR: green artificial reef. NFRC: natural fibre-reinforced concrete.

Figure 4 details the construction of the artificial reef if 100% of the frames are substituted by vegetable fibres. As can be seen, the upper parts of the artificial reef have discontinuous and short fibres randomly distributed, while the bottom part must include continuous fibres.

### 2.2. Analysis of Materials Flow

Robèrt et al. [113] indicated that “dematerialisation” is one of the mechanisms to achieve environmental sustainability, through the reduction in material flows. The implementation of CE implies converting linear flows into circular material [114]. This requires the assessment of the balance between profits and costs associated with these loops. Suarez-Eiroa et al. [38] propose the following four objectives:

(1) The first objective is to decouple the economic development of the use of finite resources [38,89,92,114,115]. This can be stated as “the extraction rate of renewable resources has to be lower than the regeneration rate of those resources” [41]. All of this is to be considered within a framework [34,35,116] that is in a constant state of development [117,118,119,120]. Consequently, it is necessary to distinguish between two types of inputs: renewable (RI) and non-renewable (NRI). It is also possible to estimate the use rate of each type of input as can be deducted from Equations (1) and (2). In accordance with the above references, the consumption rate of renewable inputs (Equation (1)) should be lower than the corresponding regeneration rate, Equation (3):

(1) $$RI_{rate}=\frac{RI}{NRI+RI}$$

(2) $$NRI_{rate}=\frac{NRI}{NRI+RI}$$

(3) $$RI_{rate}\ll RI_{regeneration\;rate}$$

where *RI* and *NRI* are the corresponding quantities of renewable and non-renewable inputs, respectively.

(2) Since non-renewable resources are running out, it is required that “the consumption of non-renewable resources must be minimised as much as possible and, eventually, disposed” [38]. Therefore, the NRI rate (Equation (2)) must be lower than the RI rate (Equation (1)), as indicated in Equation (4) [44]. In this context, Turner [121] presented a less restrictive condition. That is, non-renewable resource consumption should be as low as possible, as shown in Equation (5), giving preference to the consumption of renewables [121]:

(4) $$NRI_{rate}\leq RI_{\;rate}$$

(5) $$NRI_{rate}\to 0$$

By way of example, it is important to note that 1 m^3^ of CAR includes as renewable inputs (RI) gravel (1300 kg/m^3^) and sand (750 kg/m^3^), while cement (350 kg/m^3^) and frames (45 kg/m^3^) constitute non-renewable inputs (NRI). The previous quantities are included in Table 4. The calculation of RI and NRI rates, included in Table, 5 is based on Equations (1) and (2), respectively, i.e., *RI_rate_* = (1300 + 750)/(350 + 45 + 1300 + 750) = 0.839 and *NRI_rate_* = (350 + 45)/(350 + 45 + 1300 + 750) = 0.161. The RI and NRI rates corresponding to GAR1, GAR2, GAR3 and GAR4 were obtained similarly, but this time taking into account the corresponding substitution percentages included in Table 4. The reader should bear in mind that both the shells and the vegetable fibres are renewable inputs. The reader can find in Table 5 both the RI and NRI rates.

(3) “The rate of emission of biological wastes must be lower than the natural capacity of ecosystems to assimilate the delivered wastes” [38]. The objective of minimising waste has also been identified by other authors [38,89,90,115,122,123]. In this sense, it is important to distinguish between two types of waste: biological outputs (BOs) and technical outputs (TOs) [89]. Consequently, it is necessary to estimate the generation rates of both biological and technical outputs, Equations (6) and (7), respectively. According to Daly´s proposed operational criteria [44], the emission rate for biological waste, *BO_rate_*, must be inferior to the natural capacity of ecosystems to assimilate released wastes, Equation (8):

(6) $$BO_{rate}=\frac{BO}{BO+TO}$$

(7) $$TO_{rate}=\frac{TO}{BO+TO}$$

(8) $$BO_{rate}\ll Assimilation\;capacity\;of\;ecosystems$$

where *BO* and *TO* are the corresponding quantities of biological and technical outputs, respectively.

(4) “Technical outputs must be minimised or, eventually, eliminated” [38]. Technical waste is not biodegradable. It requires human intervention in the process of its reincorporation into the economic system. Consequently, technical waste must be minimised or even eliminated [89,124]:

(9) $$TO_{rate}\to 0$$

From the data included in Table 4, 1 m^3^ of CAR produces as biological outputs gravel (1300 kg/m^3^), sand (750 kg/m^3^) and cement (350 kg/m^3^), while frames (45 kg/m^3^) produce technical outputs. The calculation of BO and TO rates in Table 6 is based on Equations (6) and (7), respectively, i.e., *BO_rate_* = (1300 + 750 + 350)/(350 + 45 + 1300 + 750) = 0.982 and *TO_rate_* = 45/(350 + 45 + 1300 + 750) = 0.018. The BO and TO rates corresponding to GAR1, GAR2, GAR3 and GAR4 were obtained similarly (Table 6), once again, taking into account the corresponding substitution percentages included in Table 4.

To summarise, Table 7 illustrates the results for the CAR, GAR1 (the best “realistic” proposal, i.e., employing 25% eucalyptus frames) and GAR4 (“idealistic” proposal). These data are obtained from Table 5 and Table 6. For instance, regarding the CAR, *NRI_rate_* = 0.161, *RI_rate_* = 0.839 (Table 5), *BO_rate_* = 0.982 and *TO_rate_* = 0.018 (Table 6).

### 2.3. Sustainability Analysis

The MIVES (Spanish acronym of Integrated Value Model for Sustainability Assessment) method was employed to quantify the sustainability index (*SI*) of each one of the four green artificial reefs defined in Table 4 (GAR1–GAR4). A CAR, with no substitute materials, was also included in the assessment. MIVES is a deterministic multi-criteria decision making (MCDM) technique. Consequently, it constitutes a formal approach to handling complex decision-making situations, providing coherence in the decision-making processes [125,126,127].

The first step in the MIVES method consists of defining the decision tree, also known as the requirement tree. It usually consists of three levels: requirements (R), criteria (C) and indicators (I). The indicators are the most specific aspects and the requirements the most general ones. Three basic requirements were considered: economic, environmental and social. Table 8 outlines the decision tree together with the weights of the different branches. It is important to note that, in the existing literature, there are no similar models to assess the sustainability of artificial reefs. The weights, indicated between parentheses, were set by experts in the field. Several objective methods can be found in the literature to establish the weights through mathematical expressions. Nevertheless, subjective weighting criteria are most commonly used, and objective methods are particularly recommended when there is no agreement among the sets of weights proposed by the experts [128,129]. In any case, a sensitivity analysis of the values defined for the weights is carried out in Section 3.

It is necessary to make some clarifications about the model and about the way in which some indicators are evaluated. First, it is important to note than only the indicators that generate differences in the results among the alternatives should be included in a MIVES model. In other words, if all the alternatives under assessment adopt the same (or very close) input value for a specific aspect, the corresponding indicator can be removed from the model. This is the reason why some indicators such as the employment generation, the positive impact on the ecosystems or the potential recreational uses (fishing, diving, etc.) among others, are not included in Table 8.

The total cost indicator (R1C1I1) includes both the cost of the materials as well as the cost of the energy used in their production. A value of EUR 0.12/kWh was assumed for such a purpose [130]. The reader should bear in mind that the costs associated with the production and assembling processes of each AR unit, as well as the costs derived from the transportation and the positioning and anchoring, were not included in this indicator. The main reason is that the manufacturing, transport and installation costs are expected to be the same for all the alternatives included in this study. Indicator R1C2I1 serves to assess the costs that have been avoided by using the shells in the different AR units, preventing their disposal. In other words, a higher amount of shells used in an AR unit is linked to a lower amount of not exploitable waste to be eliminated (with the corresponding savings).

Indicator R2C1I1 is similar to the previous one. In fact, they are measured in the same units and through the same value function. Nevertheless, R2C1I1 takes into account the avoided generation of wastes derived from the use of shells in the production of AR units. In other words, indicator R1C2I1 assesses the economic side while indicator R2C1I1 focused on the environmental side of the “same coin”.

The amount of resources employed in each AR unit was evaluated on the basis of indicators R2C2I1–R2C2I3. A higher input value, i.e., a higher consumption, always generates a lower level of satisfaction. The consumption of shells was not included in this criterion, since the derived positive impacts were previously assessed in other indicators. The amount of water used in each alternative is always the same and this is the reason why the water use indicator was excluded. On the other hand, these three indicators (R2C2I1–R2C2I3) also served, to a certain extent, to quantify some of the most common emissions (CO_2_, CO, NO_x_, VOCs (volatile organic compounds), SO_x_ and PM_10_ (particulate matter with diameters equal to or smaller than 10 micrometres)). This has been possible because of the use of the weights (5%, 70% and 25%) based on [131]. In [131], Mel estimated the average contribution of the aggregates, cement and steel production to the previous pollutants, obtaining values in line with the ones employed in this study.

Once again, at the time of assessing the total energy consumption (R2C3I1), the manufacturing, transport and installation processes were excluded. The reason is the same that was previously explained. The generation of waste at the end of each AR’s useful life is assessed through indicators R2C4I1 and R2C4I2. Logically, a higher input value generates a lower satisfaction and vice versa.

From a social point of view, two indicators were taken into account (R3C1I1 and R3C1I2). They assessed the potential contribution of each AR unit to the sustainability in the canning and wood industries, respectively. A higher consumption of shells or fibres is assumed to generate a positive impact on the corresponding sectors. These two indicators are measured in points (from 0 to 100). The number of points for each alternative was directly estimated from the amount of shells and fibres consumed.

MIVES transforms different types of indicators, measured in different units, into a dimensionless quantity, *V_i_*, called value or level of satisfaction, through the use of value functions, Equation (10) [125,126,127]. According to this, from a specific input value (*X_i_*) measured in the corresponding units (m, EUR, kg, etc.), value functions return a value between 0 and 1, the minimum and maximum levels of satisfaction, respectively:

(10) $$V_{i}=\frac{1-exp\left(-K_{i}\cdot {\left(\frac{\left|X_{i}-X_{min}\right|}{C_{i}}\right)}^{P_{i}}\right)}{1-exp\left(-K_{i}\cdot {\left(\frac{\left|X_{max}-X_{min}\right|}{C_{i}}\right)}^{P_{i}}\right)}$$

where *X_min_* is the input value associated with the minimum level of satisfaction (0), while *X_max_* is the input value linked to the maximum level of satisfaction (1). *C_i_*, *K_i_* and *P_i_* are parameters that allow the user to define value functions with different geometries (linear, convex, concave or s-shaped). Table 9 shows the values that these parameters adopt for the different value functions as well as the units of measurement for the indicators.

Table 10 contains the model input values for the five alternatives under assessment.

With the information provided in Table 8, Table 9 and Table 10, it is possible to estimate the sustainability index (*SI*) for each alternative. In the present work, the so-called SAW (simple additive weighting) method was employed, and the *SI* is given by Equation (11) [125,126,127,132]:

(11) $$SI=\overset{N}{\underset{i=1}{\sum }}\alpha_{i}\cdot \beta_{i}\cdot \gamma_{i}\cdot V_{i}$$

where *N* is the number of indicators considered in the requirement tree (11 in this case); *α_i_*, *β_i_* and *γ_i_* are the weights for the requirements, criteria and indicators, respectively, contained in Table 8. The *SI* also falls within the interval [0,1], again the worst and best possible values, respectively.

It is important to remark that MCDM methods are affected by subjectivity and MIVES is not an exception. In fact, subjectivity comes into play at the time of establishing the weights and also at the time of defining value functions. Consequently, different sets of results can be obtained when Equation (11) is used, depending on these factors. Nevertheless, although the numerical results may change, the ranking of alternatives should be similar if reasonable weights and value functions are defined. In other words, changes in the positions can only appear in those alternatives that have very close input values and therefore, very similar performances. Similarly, the difference between the sustainability indices obtained by the two alternatives also changes if different weights and value functions are used. Nevertheless, if those differences are compared, proportionality will be maintained to certain extent. A possible solution to manage these issues is to perform a sensitivity analysis [126,127], as the one implemented in this study. Another option is to transform the model into a probabilistic one, defining probability distributions for all the parameters affected by subjectivity and applying Monte Carlo simulation [125]. Nevertheless, this is out of the scope of this study, although it will be considered as a possible future development.

## 3. Results and Discussion

The sustainability index (*SI*) obtained by Equation (11) can be divided into three partial sub-indices, each one related to a specific sustainability dimension [127]. In other words, the MIVES method allows the user to obtain the contribution that each alternative makes to economic, environmental and social pillars. Therefore, it is also possible to calculate an economic index (*SI_Economic_*), an environmental index (*SI_Environmental_*) and a social index (*SI_Social_*). Equation (11) is also used for the estimation of these indices but only taking into account the indicators and the criteria that belong to each dimension [127]. Table 11 shows the sustainability index (*SI*) as well as the partial sub-indices for all the alternatives. The numbers in brackets indicate the position that each alternative occupies in a descending ranking (from best to worst).

As can be seen in Table 11, the alternatives always occupy the same position in the different rankings. As expected, GAR4 obtained the best results. As indicated previously, in this alternative, both a small percentage of cement and sand was substituted by shells. Something similar happens in the cases of alternatives GAR1, GAR2 and GAR3. Nevertheless, the main difference among the green alternatives lies on the amount of steel used since GAR4 replaces all the steel by eucalyptus fibres. This leads to the total elimination of technical wastes and the optimisation of biological ones. However, as indicated previously, this is an “idealistic” option that must be carefully analysed through mechanical and durability tests, confirming its adequacy.

On the other hand, the conventional alternative (CAR) is always located at the bottom of the different rankings. From an economic point of view, it is important to remark that conventional materials (cement, sand and steel) are more expensive than the corresponding substitutes (shells and eucalyptus fibres). At the same time, the non-use of shells does not reduce the generation of wastes from the canning industry, which has a negative impact from both the environmental and social points of view. For the remaining alternatives, GAR2 resulted to be the best “realistic” option (between CAR, GAR1, GAR2 and GAR3). This represents 20% of the sand being replaced by shells (150 kg) and 25% of steel frames replaced by fibres (11.25 kg). GAR1 and GAR3 also present a 25% of replacement in terms of steel. However, the substitution of sand and cement by shells is lower in both cases (35 kg for GAR1 and 92.5 kg for GAR3). In fact, the higher use of seashells in GAR3 explains why it over performed GAR1. In conclusion, a higher consumption of seashells and fibres instead of conventional materials should lead to better results in terms of sustainable development.

A sensitivity analysis was carried out with the objective of testing the robustness of the model. In particular, six additional cases regarding the weights of the requirements were considered (Table 12).

As can be seen in Table 12, the numerical values for the sustainability indices (*SIs*) suffered slight changes. However, what is important is to analyse the position that each alternative occupies in the corresponding rankings. In this regard, the positions remain unchanged. In other words, GAR4 is always the best option followed by GAR2, GAR3, GAR1 and CAR, respectively. Consequently, it is possible to say that the model is robust since small changes in the weights do not significantly affect the results. Small variations in other weights as well as in the value functions parameters should not considerably alter the ranking.

**Figure 5:**
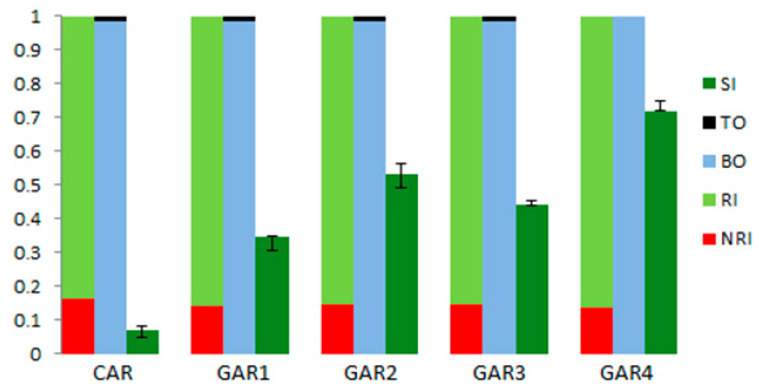
Graphical representation of the results obtained in terms of both materials flow and sustainability analyses. SI: sustainability index; TO: technical output rate; BO: biological output rate; RI: renewable input rate; NRI: non-renewable input rate; CAR: conventional artificial reef;. GAR1: 10% of cement replaced by shells and 25% of steel substituted by eucalyptus; GAR2: 20% of sand replaced by shells and 25% of substitution for the steel; GAR3: 5% of cement and 10% of sand replaced by shells; 25% of substitution for the steel; GAR4: 5% and 10% of substitution for cement and sand, 100% of replacement for steel.

Finally, it is interesting to compare the main results obtained with the two methodologies used in this study. The reader can find in Figure 5 a graphic representation of the results in terms of both materials flow and sustainability. The bars in dark green represent the sustainability index (SI) of each alternative, obtained using Equation (11). The minimum and maximum sustainability indices derived from the sensitivity analysis are also included in the same bars (lines in black colour). The rates of renewable (RI) and non-renewable resources (NRI) employed in each alternative are depicted in light green and red bars, respectively; they are the result of using Equations (1) and (2). As can be seen, the sum of the renewable (RI) and non-renewable (NRI) rates is always equal to 1. Similarly, the rates for the biological (BO) and technical (TO) outputs (wastes) associated with each alternative are shown in the blue and black bars, respectively. Once again, the sum of both rates is equal to 1. The rates for the outputs are the result of using Equations (6) and (7).

The results of CE application, with the substitution of cement and sand with processed shells, combined with the substitution of metal frames with eucalyptus fibres, leads to an increase in both RI and BO rates. These rates decrease slightly in cases where aggregates are used as a cement substitute. From the results obtained by using the material flow analysis, GAR1 can be considered as the most advantageous of the “realistic” alternatives since it obtained the highest RI rate, as indicated in Table 5. However, taking into consideration the *SIs* provided by the MIVES model, which jointly considers economic, environmental and social issues, GAR2 appeared to be the most appropriate of the “realistic” options due to the reasons indicated above. On the other hand, both methodologies confirmed that the “idealistic” option, GAR4, was the most adequate one. Furthermore, it is possible to say that the proposed MIVES model is more sensitive than the first methodology at the time of comparing the performance of different alternatives.

## 4. Conclusions

The production of artificial reefs through the composition of waste combined with reinforced concrete can be of interest provided that the incorporated materials are environmentally admissible. In practice, this gives birth to green artificial reefs (GARs), based on using renewable and biological material flows; designed to be reintroduced smoothly into the biosphere with a resulting reduction in technical wastes.

Consequently, in this manuscript, two different methodologies were applied with the objective of assessing the potential contribution of different GARs to both the circular economy and sustainability. The first one is based on materials flow while the second relies on the MIVES method. Different percentages of substitution were proposed for cement, sand and metal frames with mussel shells and eucalyptus vegetable fibres, resulting in four green artificial alternatives. A conventional artificial reef was also considered, since it serves as a reference for comparison. To the best of the authors’ knowledge, this is the first time that a study like this one was been carried out.

The most important findings of this study are:

- There are important differences in the contribution to CE and to sustainability between green artificial reefs and the conventional alternative. In fact, according to the first methodology, a partial substitution of cement and sand with processed shells and the total substitution of metal frames with eucalyptus fibres (GAR4) is the most advantageous outcome. The second methodology also assigned the highest performance to the same alternative.
- From the results obtained with the MIVES model, it is possible to conclude that the contribution to sustainable development increases if cement or sand are substituted by seashells. The higher the amount of substitution, the higher the sustainability index will be.
- The MIVES model resulted to be robust, since the sensitivity analysis did not affect the position that each alternative occupies in the ranking of performance. In other words, despite the fact that the MIVES models are affected by subjectivity, small modifications in the model does not generates changes in the ranking of alternatives.
- Green artificial reefs resulted to be of significant interest for Galicia, a region with the second biggest canning industry in the world. The use of shells as a partial substitute of both cement and sand will serve to manage part of the shell waste generated every year in Galicia. In this sense, the present work provides a framework for the transition from conventional artificial reefs to green artificial reefs.
- Green artificial reefs could also be of great interest in other regions with similarities to Galicia.

Future works must be aimed to analyse the performance and durability of shells and vegetable fibres as a component of marine concrete structures, since this application is still in an incipient stage. Furthermore, the MIVES model can also be improved in several ways: (i) increasing the number of indicators or (ii) including a methodology to manage the uncertainty and the subjectivity such as Monte Carlo simulation or fuzzy logic, among other options.

## Figures and Tables

**Table 1:** Potential effectiveness of artificial reefs (ARs) in terms of economic, environmental and social objectives. Expected results that should be demonstrated by using scientific methodologies.

AR Type	Underlying Principle	Potential Degree of Compliance with Economic, Environmental and Social Objectives		
		Economic	Environmental	Social
Sinking disused vessels	Utilising waste	High	Low	Mid-level
^1^ OAR	Utilising waste	High	Null	Low
^2^ CAR	Linear economy	High	Mid-level	Mid-level
^3^ GAR	Circular economy	Mid-level	High	High

^1^ opportunity artificial reef, ^2^ conventional artificial reef, ^3^ green artificial reef.

**Table 2:** The comparison of mechanical properties of several natural and artificial fibres [84,85].

Fibre	Specific Gravity (Kg/M^3^)	Water Absorption (%)	Tensile Strength (Mpa)	Modulus of Elasticity (Gpa)
Polypropylene	913	0	250	2.0
PVA F-45	1300	0	900	23
Sisal	1370	110	347–378	15.2
Bamboo	1158	145	73–505	10–40
Eucalyptus	760	50	48–181	6.5–33

**Table 3:** CAR components as raw materials, services and chemical or organic residues.

AR Components: Raw Materials, Services and Waste Products Generated	Environmental Impact		Action Taken within CE ^1^ Framework
	Raw Materials (Production, Exploitation, Service)	Residues	
Cement	High (production)	Organic	Partial substitution
Aggregates (sand + gravel)	Mid-range (exploitation)	Organic	Partial substitution
Gravel	Low (exploitation)	Organic	-
Metal frames	High (production)	Chemical	Partial or complete substitution
Formwork moulds	Mid-level (production)	Chemical	Re-usable variety
Land and sea transport	Mid-level (service)	Chemical	Logistic analysis
Other: - Fluidifying additives - Water	Low	Organic	-

^1^ circular economy

**Table 4:** Proposals analysed in the present work. Materials with percentage substitution and reference.

Proposal	Base Material	Dosage kg/m^3^	Substitution (% Weight)	Substitution Materials	Reference
CAR ^1^	Cement	350	-	-	-
	Sand	750	-	-	-
	Gravel	1300 ^6^	-	-	-
	Frames	45 ^7^	-	-	-
GAR1 ^2^	Cement	350	10%	Oyster and mussel shells crushed and calcined at 600 °C	[99]
	Sand	750	-	-	-
	Gravel	1300 ^6^	-	-	-
	Frames	45 ^7^	25%	Random vegetable fibres (eucalyptus)	[68]
GAR2 ^3^	Cement	350	-	-	-
	Sand	750	20%	Oyster and mussel shells crushed and baked at 300 °C	[16]
	Gravel	1300 ^6^	-	-	-
	Frames	45 ^7^	25%	Random vegetable fibres (eucalyptus)	[68]
GAR3 ^4^	Cement	350	5%	Oyster and mussel shells crushed and calcined at 600 °C	[99]
	Sand	750	10%	Oyster and mussel shells crushed and baked at 300 °C	[16]
	Gravel	1300 ^6^	-	-	-
	Frames	45 ^7^	25 %	Random vegetable fibres (eucalyptus)	[68]
GAR4 ^5^	Cement	350	5%	Oyster and mussel shells crushed and calcined at 600 °C	[99]
	Sand	750	10%	Oyster and mussel shells crushed and baked at 300 °C	[16]
	Gravel	1300 ^6^	-	-	-
	Frames	45 ^8^	100%	Vegetable fibres: random and in bars (eucalyptus)	[68]

^1^ Conventional artificial reef made from reinforced concrete; ^2^ 10% of the cement is substituted by shells and 25% of the steel frame is substituted by eucalyptus fibres; ^3^ 20% of the sand is substituted by shells and 25% of the steel frame is substituted by eucalyptus fibres; ^4^ 5% of the cement is substituted by shells, 10% of the sand is substituted by shells and 25% of the steel frame is substituted by eucalyptus fibres; ^5^ 5% of the cement is substituted by shells, 10% of the sand is substituted by shells and 100% of the steel frame is substituted by eucalyptus fibres.^6^ Gravel extracted from gravel pits or crushing in quarries, ^7^ corresponds to a reinforced slab of 80 kg/m^3^ and a top part with a reinforcement of 35 kg/m^3^, ^8^ use of eucalyptus vegetable fibres arranged randomly or in bars.

**Table 5:** Non-renewable inputs (NRI) and renewable inputs (RI) rates for the five artificial reefs analysed in this study.

Parameter	CAR ^1^	GAR1 ^2^	GAR2 ^3^	GAR3 ^4^	GAR4 ^5^
*NRI_rate_*	0.161	0.143	0.145	0.148	0.135
*RI_rate_*	0.839	0.857	0.855	0.852	0.865

^1^ Conventional artificial reef made by reinforced concrete; ^2^ 10% of the cement is substituted by shells and 25% of the steel frame is substituted by eucalyptus fibres; ^3^ 20% of the sand is substituted by shells and 25% of the steel frame is substituted by eucalyptus fibres; ^4^ 5% of the cement is substituted by shells, 10% of the sand is substituted by shells and 25% of the steel frame is substituted by eucalyptus fibres; ^5^ 5% of the cement is substituted by shells, and 10% of the sand is substituted by shells and 100% of the steel frame is substituted by eucalyptus fibres.

**Table 6:** Biological output (BO) and technological output (TO) rates for the five artificial reefs analysed in this study.

Parameter	CAR ^1^	GAR1 ^2^	GAR2 ^3^	GAR3 ^4^	GAR4 ^5^
*BO_rate_*	0.982	0.987	0.987	0.987	1
*TO_rate_*	0.018	0.013	0.013	0.013	0

^1^ Conventional artificial reef made by reinforced concrete; ^2^ 10% of the cement is substituted by shells and 25% of the steel frame is substituted by eucalyptus fibres; ^3^ 20% of the sand is substituted by shells and 25% of the steel frame is substituted by eucalyptus fibres; ^4^ 5% of the cement is substituted by shells, 10% of the sand is substituted by shells and 25% of the steel frame is substituted by eucalyptus fibres; ^5^ 5% of the cement is substituted by shells, 10% of the sand is substituted by shells and 100% of the steel frame is substituted by eucalyptus fibres.

**Table 7:** Input and output results for CAR, GAR1 and GAR4.

Parameter	CAR ^1^	GAR1 ^2^	GAR4 ^3^
*NRI_rate_*	0.161	0.143	0.135
*RI_rate_*	0.839	0.857	0.865
*BO_rate_*	0.982	0.987	1
*TO_rate_*	0.018	0.013	0

^1^ Conventional artificial reef made by reinforced concrete; ^2^ 10% of the cement is substituted by shells and 25% of the steel frame is substituted by eucalyptus fibres; ^3^ 5% of the cement is substituted by shells, 10% of the sand is substituted by shells and 100% of the steel frame is substituted by eucalyptus fibres.

**Table 8:** Decision tree of the sustainability model.

Requirements (α_i_)	Criteria (β_i_)	Indicators (γ_i_)
R1. Economic (21%)	R1C1. Total cost (82%)	R1C1I1. Total cost (100%)
	R1C2. Influence on the canning industry (18%)	R1C2I1. Reduction in wastes (shells) (100%)
R2. Environmental (45%)	R2C1. Influence on the canning industry (28%)	R2C1I1. Reduction in wastes (shells) (100%)
	R2C2. Material resources consumption and emissions (36%)	R2C2I1. Aggregates (5%)
		R2C2I2. Cement (70%)
		R2C2I3. Steel frame (25%)
	R2C3. Energy consumption (24%)	R2C3I1. Total energy consumption (100%)
	R2C4. Generation of wastes (12%)	R2C4I1. Steel frames (25%)
		R2C4I2. Concrete (75%)
R3. Social (34%)	R3C1. General prosperity (100%)	R3C1I1. Sustainability on the canning industry (62.5%)
		R3C1I2. Sustainability on the wood industry (37.5%)

**Table 9:** Parameters for the value functions.

Indicators	*X_min_*	*X_max_*	*P_i_*	*K_i_*	*C_i_*	Trend	Shape
R1C1I1 (EUR/AR unit)	250	175	1	0.01	250	Decreasing	Linear
R1C2I1 (kg/AR unit)	0	150	1	0.01	150	Increasing	Linear
R2C1I1 (kg/AR unit)	0	150	1	0.01	150	Increasing	Linear
R2C2I1 (kg/AR unit)	2050	1900	1	0.01	2050	Decreasing	Linear
R2C2I2 (kg/AR unit)	350	300	1	0.01	350	Decreasing	Linear
R2C2I3 (kg/AR unit)	45	0	1	0.01	45	Decreasing	Linear
R2C3I1 (kWh/AR unit)	700	550	1	0.01	700	Decreasing	Linear
R2C4I1 (kg/AR unit)	50	0	1	0.01	50	Decreasing	Linear
R2C4I2 (kg/AR unit)	2650	2600	1	0.01	2650	Decreasing	Linear
R3C1I1 (points, based in the amount of shells employed)	0	100	1	0.01	100	Increasing	Linear
R3C1I2 (points, based in the amount of fibres employed)	0	100	1	0.01	100	Increasing	Linear

**Table 10:** Model input values for the different alternatives.

Indicator	Alternatives				
	CAR ^1^	GAR1 ^2^	GAR2 ^3^	GAR3 ^4^	GAR4 ^5^
R1C1I1 (EUR/AR unit)	243.79	224.60	227.15	225.87	184.04
R1C2I1 (kg/AR unit)	0	35	150	92.5	92.5
R2C1I1 (kg/AR unit)	0	35	150	92.5	92.5
R2C2I1 (kg/AR unit)	2050	2050	1900	1975	1975
R2C2I2 (kg/AR unit)	350	315	350	332.5	332.5
R2C2I3 (kg/AR unit)	45	33.75	33.75	33.75	0
R2C3I1 (kWh/AR unit)	692	640.81	673.31	657.06	555.98
R2C4I1 (kg/AR unit)	45	33.75	33.75	33.75	0
R2C4I2 (kg/AR unit)	2600	2611.25	2611.25	2611.25	2645
R3C1I1 (points, based in the amount of shells employed)	0	23.32	100	61.66	61.66
R3C1I2 (points, based in the amount of fibres employed)	0	25	25	25	100

^1^ Conventional artificial reef made by reinforced concrete; ^2^ 10% of the cement is substituted by shells and 25% of the steel frame is substituted by eucalyptus fibres; ^3^ 20% of the sand is substituted by shells and 25% of the steel frame is substituted by eucalyptus fibres; ^4^ 5% of the cement is substituted by shells, 10% of the sand is substituted by shells and 25% of the steel frame is substituted by eucalyptus fibres; ^5^ 5% of the cement is substituted by shells, 10% of the sand is substituted by shells and 100% of the steel frame is substituted by eucalyptus fibres.

**Table 11:** MIVES (Spanish acronym of Integrated Value Model for Sustainability Assessment) results for the alternatives under assessment.

Alternatives	Results			
	*SI_Economic_*	*SI_Environmental_*	*SI_Social_*	*SI*
CAR ^1^	0.0680 (5)	0.1058 (5)	0 (5)	0.0619 (5)
GAR1 ^2^	0.3201 (4)	0.4389 (4)	0.2404 (4)	0.3465 (4)
GAR2 ^3^	0.4301 (2)	0.4429 (2)	0.7191 (2)	0.5341 (2)
GAR3 ^4^	0.3753 (3)	0.4411 (3)	0.4802 (3)	0.4406 (3)
GAR4 ^5^	0.8325 (1)	0.6297 (1)	0.7611 (1)	0.7170 (1)

^1^ Conventional artificial reef made by reinforced concrete; ^2^ 10% of the cement is substituted by shells and 25% of the steel frame is substituted by eucalyptus fibres; ^3^ 20% of the sand is substituted by shells and 25% of the steel frame is substituted by eucalyptus fibres; ^4^ 5% of the cement is substituted by shells, 10% of the sand is substituted by shells and 25% of the steel frame is substituted by eucalyptus fibres; ^5^ 5% of the cement is substituted by shells, 10% of the sand is substituted by shells and 100% of the steel frame is substituted by eucalyptus fibres.

**Table 12:** Sustainability indices (*SIs*) derived from the sensitivity analysis.

Weights and Alternatives	Cases					
	Case 1	Case 2	Case 3	Case 4	Case 5	Case 6
R1. Economic	21%	34%	34%	45%	45%	33.33%
R2. Environmental	34%	21%	45%	21%	34%	33.33%
R3. Social	45%	45%	21%	34%	21%	33.33%
CAR ^1^	0.0503 (5)	0.0453 (5)	0.0707 (5)	0.0528 (5)	0.0666 (5)	0.0579 (5)
GAR1 ^2^	0.3246 (4)	0.3092 (4)	0.3568 (4)	0.3180 (4)	0.3438 (4)	0.3332 (4)
GAR2 ^3^	0.5645 (2)	0.5628 (2)	0.4965 (2)	0.5310 (2)	0.4951 (2)	0.5307 (2)
GAR3 ^4^	0.4449 (3)	0.4363 (3)	0.4270 (3)	0.4248 (3)	0.4197 (3)	0.4322 (3)
GAR4 ^5^	0.7314 (1)	0.7578 (1)	0.7262 (1)	0.7656 (1)	0.7486 (1)	0.7411 (1)

^1^ Conventional artificial reef made by reinforced concrete; ^2^ 10% of the cement is substituted by shells and 25% of the steel frame is substituted by eucalyptus fibres; ^3^ 20% of the sand is substituted by shells and 25% of the steel frame is substituted by eucalyptus fibres; ^4^ 5% of the cement is substituted by shells, 10% of the sand is substituted by shells and 25% of the steel frame is substituted by eucalyptus fibres; ^5^ 5% of the cement is substituted by shells, 10% of the sand is substituted by shells and 100% of the steel frame is substituted by eucalyptus fibres.
